# Utilization of HCV viremic donors in kidney transplantation: a chance or a threat?

**DOI:** 10.1080/0886022X.2022.2047069

**Published:** 2022-03-09

**Authors:** Paulina Czarnecka, Kinga Czarnecka, Olga Tronina, Teresa Baczkowska, Magdalena Durlik

**Affiliations:** Department of Transplant Medicine, Nephrology and Internal Diseases, Medical University of Warsaw, Warsaw, Poland

**Keywords:** Renal transplantation, hepatitis C, antiviral drugs, organ donors

## Abstract

Kidney transplantation is the treatment of choice in end-stage renal disease. The main issue which does not allow to utilize it fully is the number of organs available for transplant. Introduction of highly effective oral direct-acting antivirals (DAAs) to the treatment of chronic hepatitis C virus infection (HCV) enabled transplantation of HCV viremic organs to naive recipients. Despite an increasing number of reports on the satisfying effects of using HCV viremic organs, including kidneys, they are more often rejected than those from HCV negative donors. The main reason is the presence of HCV viremia and not the quality of the organ. The current state of knowledge points to the fact that a kidney transplant from an HCV nucleic acid testing positive (NAT+) donor to naive recipients is an effective and safe solution to the problem of the insufficient number of organs available for transplantation. It does not, however, allow to draw conclusions as to the long-term consequence of such an approach. This review analyzes the possibilities and limitations of the usage of HCV NAT + donor organs. **Abbreviations:** DAA: direct-acting antivirals; HCV: hepatitis C virus; NAT: nucleic acid testing; OPTN: Organ Procurement and Transplantation Network; KDIGO: Kidney Disease: Improving Global Outcomes; Ab: antigen; eGFR: estimated glomerular filtration rate; D: donor; R: recipient; CMV: cytomegalovirus; HBV: hepatitis B virus; UNOS: United Network for Organ Sharing; PHS: Public Health Service; EBR/GZR: elbasvir/grazoprevir; SVR: sustained virologic response; RAS: resistance-associated substitutions; SOF: soforbuvir; GLE/PIB: glecaprevir/pibrentasvir; ACR: acute cellular rejection; AR: acute rejection; DSA: donor-specific antibodies; KTR: kidney transplant recipients; AASLD: American Association for the Study of Liver Disease; IDSA: Infectious Diseases Society of America; PPI: proton pump inhibitors; CKD: chronic kidney disease; GN: glomerulonephritis; KAS: The Kidney Allocation system

## Introduction

Kidney transplantation is the treatment of choice for patients with end-stage renal disease. It not only reduces mortality in this group of patients but also improves their quality of life. However, the demand for transplant organs far exceeds their availability. According to data published by the Organ Procurement and Transplantation Network (OPTN), more than 90,000 people are awaiting a kidney transplant [1]. Importantly, dialysis-dependent patients have a higher mortality rate compared to general population which is mostly attributable to cardiovascular disease [2]. As a result, many of kidney transplant candidates will not survive until they obtain a transplant [3]. This is the result of a long waiting time for an organ, which in the US can extend to 3–5 years [4].

Many steps are being taken to increase the organ donor pool including living donors and donors after cardiac death. Despite this, demand for organs still exceeds the supply. Another step taken in order to resolve the organ shortage issue is also the use of organs from donors infected with the hepatitis C virus (HCV). The advent of new, highly effective interferon-free therapies with an efficacy exceeding 95% allowed to utilize HCV-viremic organs especially in the setting of significant increase in mortality resulting from drug overuse, opioids in particular, in the US [5]. These donors have a higher prevalence of HCV viremia compared to standard risk criteria donors. Donors infected with HCV are usually younger than HCV-negative donors, and this can result in fewer comorbidities and improved quality of the organ, as manifested by the Kidney Donor Profile Index (KDPI) [6–9]. The use of organs from HCV donors with active viremia could also increase the donor pool of kidneys available for transplantation by as many as 500 organs per year [10].

In the past, HCV infection in a donor was considered a contraindication for kidney transplantation owing to the risk of long-term immunosuppression in the recipient. According to the Kidney Disease: Improving Global Outcomes (KDIGO) guidelines, since the 1990s, kidneys from HCV antibody-positive (Ab+) donors have been used as transplants under the condition that the recipient is also HCV Ab+. However, owing to the difficulty in differentiating donors with and without active viremia and lack of highly effective treatments, the use of such organs was limited [11]. The current guidelines of the American Society of Transplantation do not forbid the transplantation of infected organs to recipients not infected with HCV, but they indicate the necessity for further research on the consequence of such a practice [12].

This study focuses not only on the possibilities presented by the usage of HCV-viremic donor organs based on current medical knowledge, but also on additional aspects that limit the usage of such donor organs.

## Underutilization

In the past, HCV-positive organs were discarded nearly three times more often than HCV-negative ones were [13]. Between 2005 and 2014, although 6456 kidneys from HCV-seropositive donors were available for transplantation, only 37% of them were actually transplanted [10]. However, only approximately 16% of them would have been HCV viremic.

Data from the UK indicate that in as many as 76% of cases, the only cause for discarding the organ was the serological status of the donor, and only 8.9% were discarded because of the unsatisfactory condition of the organ itself [14]. This could have resulted from the fear of transmitting HCV infection as well as the findings of studies showing worse results for transplants from HCV-positive donors to HCV-negative recipients. However, such data are from a period when the treatment of HCV was purely interferon-based. In their retrospective data analysis of the OPTN registries, La Hoz et al. compared the results of kidney transplantation from HCV NAT + and HCV Ab+/NAT− donors in relation to those from HCV-negative donors [15]. They reported better kidney function as manifested by estimated glomerular filtration rate (eGFR) within 6 months after transplantation, a comparable 12-month graft survival rate, and lower frequency of acute rejection (AR) events.

The tendency to discard HCV-positive organs is visibly changing. Within the first 3 months of 2019, 200 kidneys were transplanted into HCV-negative recipients from HCV NAT + donors [16]. However, the odds of discarding HCV-viremic kidneys was 48% higher than the odds for discarding HCV-negative kidneys [17]. The latest survey on US transplant programs revealed that 58% of transplant centers offered transplantation from an HCV-viremic donor to an HCV-negative recipient (HCV D+/R-) [18]. The authors reported difficulties in insurance coverage as the primary factor that hindered the utilization of HCV-viremic kidneys in naïve recipients. Anderson et al., in turn, reported that the most discouraging factor was the risk of virus transmission (59%) and, most probably, the fear of possible complications; however, in 14% of cases, the main factor was the risk of being sued and in 3% of cases, it was a previous negative experience [19].

Until recently, HCV-viremic organs were offered to HCV-negative recipients only within clinical trials, for a carefully selected group of patients with guaranteed access to direct antiviral agents (DAAs). Significant shortening of the time spent on a waiting list and encouraging outcomes of HCV NAT D+/R- transplantations translated to the utilization of this practice as a standard of care by 14% of transplant centers according to the latest national survey [18].

The approach to HCV NAT D+/R− transplantation varies across European countries and depends on the national organ transplant program. Recommendations regarding HCV NAT testing are not unified and vary on other continents. In the authors’ country, HCV NAT D+/R− transplantation are against the national law; hence, HCV-seropositive organs are offered solely to HCV-positive recipients and a separate consent is required. Presence of viremia is verified after transplantation.

The underutilization of HCV NAT + organs can seem surprising when compared to the utilization of organs with the risk of transmission of viruses such as hepatitis B virus (HBV) or cytomegalovirus (CMV), which, despite the introduction of appropriate treatment, can result in *de novo* hepatitis and a higher risk of death after transplantation, an increased risk of rejection, or opportunistic infections by HBV and CMV [20–22] Despite this, both CMV D+/R− and anti-HBc + D+/R− organs are accepted for transplantation because of the larger survival benefits for the patient than the risks resulting from the transmission of the virus. This underscores a need for raising awareness of the HCV- viremic organs utilization and efficacy of DAAs.

## HCV testing and risk of transmission

The attitude toward an HCV-positive donor has completely changed with the introduction of obligatory testing for HCV RNA (NAT) as part of HCV infection diagnostics, in addition to the serological tests mandated by the United Network for Organ Sharing (UNOS) for potential organ donors in US [12]. This allowed the identification of donors with active viremia (HCV Ab+/NAT + or HCV Ab-/NAT+) and those without it (HCV NAT-). Such differentiation of donors is key because not every HCV-positive donor poses a risk of virus transmission through transplantation.

HCV Ab+/NAT- donors are those in whom the virus has been eradicated spontaneously or through antiviral treatment. Spontaneous eradication of the virus is possible in up to 45% of cases, but non-immunocompetent patients have a smaller chance for spontaneous eradication [23,24]. Kidney transplantation from an HCV donor (Ab+/NAT-) to an HCV-negative recipient can result in seroconversion, but the risk of viremia is minimal. Although the risk of viral transmission is low, screening for HCV viremia is advisable in an early posttransplant period.

In contrast, HCV NAT + donors, regardless of their serological status, are patients with active viremia and account for 4.2% of donors in the US [25]. HCV viremia in the donor is associated with a high risk of virus transmission. Viremia always develops in recipients after HCV D+/R- NAT liver transplants, and almost always in the case of heart, lung, or kidney transplants [26–31].

The introduction of routine HCV NAT testing helped reduce the diagnostic window, in which HCV infection is present but cannot yet be identified, from to 2–6 months for serological assays to 5–7days for NAT, thereby limiting the risk of unintentional virus transmission from an organ donor. Nonetheless, the nonintentional transmission of the virus from the donor to the recipient persists, especially in the case of donors meeting the US Public Health Service (PHS) criteria. Despite a negative serological result, the residual risk ranges from 0.26 to 300.6 for every 10000 donors and ranges from 0.027 to 32.4 for every 10000 donors when NAT is utilized [32]. Suryaprasad et al. described three cases of nonintentional HCV transmission despite a negative NAT result for the donor [33]. In this case, majority of the recipients were infected with HCV through the transplanted organ. Interestingly, at least three of the 12 recipients did not develop an HCV infection. This could point to the existence of factors other than the transplanted organ being responsible for the infection, but none have yet been identified.

The current guidelines of the US PHS have further recommendations on how to minimize the risk related to the utilization of organs from PHS criteria donors. The recommendation is to test all donors for human immunodeficiency virus, HCV, and HBV infections using NAT [34].

## Kidney transplantation from HCV-viremic donors to HCV-aviremic recipients

The efficacy of HCV NAT D+/R- kidney transplantation has been reported in several clinical trials [35–40]. The first prospective studies assessing the efficacy and safety of utilizing organs from HCV-infected donors appeared in 2017. In the THINKER study and its continuation, 20 HCV-naïve kidney transplant recipients (KTRs) received HCV-viremic organs [41,42]. Viral transmission was observed in 100% of patients. Elbasvir/grazoprevir (EBR/GZR) was initiated as soon as viremia was detected (i.e. on average 3 days after transplantation) and continued for 12 weeks. This enabled a sustained virologic response (SVR) in all the patients, and allograft function after 6 and 12 months did not vary from that of matched recipients of HCV-negative grafts. Notably, KTRs were tested for their genotype and resistance-associated substitutions (RAS) prior to randomization, and only genotypes 1 and 4 were acceptable. Those with genotype 1 were treated using DAAs for 16 weeks, and ribavirin was added as needed (Table 1).

**Table 1.** KT from HCV-viremic donors to HCV-negative recipients.

Study	Genotype	Treatment strategy	Waitlist time from consent to KT	Number of KTRs	Prevalence of detected viremia in KTRs	Time to DAAs initiation	Immunosuppression	SVR	Outcomes
Sise et al. [39]	All 6 donors had 1a genotype	Single dose preoperativel*y* + 12 weeks post-transplant: EBZ/GPR (+RBV and 16 weeks if NS5A RAS occurred)	6.5 months	8	88%	Preemptive	Induction: rATG 88% Basiliximab Maintenance immunosuppression: Tac MMF prednisone	100%	Mean Cr at 6 months 1,27mg/dl −100% patient survival −88% graft survival 12.5 % BKV 37.5% DGF 37.5% elevation in transaminases median KDPI 31% median VL_peak_ on POD 7was 69 IU/ml
Graham et al. [75]	Donor Genotype: 1 3/30 1a 18/30 1/3a 1/30 2 1/30 3 4/30 4 1/30 Unknown 2/30	Introduced postoperatively and continued for 12 weeks: GLE/PIB 29/30 SOF/VEL 1/30	1355 D	30	100%	POD 9	Induction: Basiliximab 60% rATG 26.7% rATG/IVIG Maintenance immunosuppression: Tac MMF prednisone	100%	Median eGFR: 55.5 mL/min/1.73 m2 at 6 months patient and graft survival were 100%, The median follow-up of 10 months 6,6% AR 27% DGF 3,3% CMV viremia 6,6% AST and AL*T* > 3x the ULN. KDPI: UNOS allocation 62,8% KDPI *sans* HCV 39,5% Median VL_peak_ 3.4 × 10^5^ copies/μL on POD 7 Median D of viremic detection POD2 HCV_Clearance_ on POD 53
Feld et al. [36]	Donor genotypes: 1a 7/18 1 b 1/18 2 2/18 3 5/18 2/18 unknown unspecified 1/18	Single dose preoperativel*y* + 7 Ds post-transplant: Ezetimibe + GLE/PIB	KTRs were given 3-12 h prior to transplantation to sign the ICF	10 (13 lungs 6 harts 1 kidney-pancreas)	60%	preemptive		100%	Median follow-up 36 weeks Grade 3 ALT elevation No AR episodes in KTRs 100% graft and patient survival among KTRs Median VL_peak_ on POD 1 1.87 log_10_ IU/mL
Terrault et al. PROACT [35]	Not provided	Introduced postoperatively and continued for 12 weeks: SOF/VEL	48 D	11 KTRs	91%	POD 16.5	Induction: rATG 82% Basiliximab	100%	100% patient and graft survival 18% DGF 9% NASH 18% elevation of LFTs median KDPI 52% median VL on POD 3 was 3.6 log_10_ IU/mL
Crismale et al. [84]	KTRs Genotypes (obtained after testing positive for HCV RNA): 4/13 1a 2/13 1 b 5/13 3	Introduced postoperatively and continued for 12 weeks: SOF/VEL 55% SOF/LED 27% GLE /PIB 18%	79 D	13	85%	POD 40	Induction: rATG 92% Basiliximab Maintenance immunosuppression: Tac MMF prednisone	100%	-Median eGFR: 69.9 mL/min/1,73m^2^ 100% patient and graft survival median follow-up 8 months 18% flu-like symptoms, fatigue, malaise KDPI 52% median VL at POW 1 was 56 173 IU/L median VL at DAAs initiation was 8 661 412 IU/L
Goldberg et al. [41] Thinker	GT 1 10/10	Introduced postoperatively and continued for 12 weeks: EBZ/GPR +/− RBV (if NS5A RAS occurred 16 weeks)	58 D	10	100%	POD 3	Induction: rATG 92% Maintenance immunosuppression: Tac MMF prednisone	100%	Median Cr 1.1 mg/dL at 6 months, 62.8 mL/min/1.73 m2, median follow-up 6 months 1/10 DGF 1/10 proteinuria due to FSGS •2/10 transient elevation in transaminases median KDPI 42%
Reesee et al.[42] Thinek-2	Donor GT: GT 1 100%	Introduced postoperatively and continued for 12 weeks: EBZ/GPR (16 weeks + RBV if NS5A RAS occurred)	57 D	20	100%	POD3	Induction: rATG Maintenance immunosuppression: Tac MMF prednisone	100%	eGFR at 6 months 67.5 mL/min/1.73 m2, at 12 months 72.8 mL/min/1.73 m2 3/20 developed dnDSA 1/20 proteinuria due to FSGS 5/20 transient elevation in transaminases (THINKER-1) 5/20 DGF No acute rejection episodes Median KDPI UNOS 46%
Gupta et al.[43] **DAPPeR** **REFORM HEPC**	Donor Genotype available in 27/50: 19/31 1a 2/31 2 6/31 3	Pre-transplant: - SOF/VEL x 1 dose Post-transplant: - Group 1 - SOF/VEL 1 dose - Group 2 SOF/VEL 3 doses - If KTR tested positive for HCV NAT, treated with - Group 1: EBR/GZR 12 weeks (GT1) - Group 2 A: SOF/VEL +/- RBV 12 weeks (GT 2,3) - Group 2B treatment based on genotype and resistance testing	30D	50	−34% had detectable viremia post-transplant −12% required treatment Group1 30% Group2 3/40 7,5%(group 2 A 2/15 13%, group 2B 1/25 4%)	preemptive	Induction: rATG Maintenance immunosuppression: Tac MMF prednisone	83%	Mean +/-(SD) eGFR: 58 mL/min/1,73m^2^ •98% patient and graft survival Median follow-up 8 months 2/5 required 2^nd^ line treatment 6^th^ patient failed 2^nd^ line DAA ACR 4% 4% transient elevation in transaminases (KTRs without viremie detected) Dn DSA 6% DGF 48% KDPI mean SD: -UNOS allocation 62+/-18 ‘optimal’ 37 +/- 18
Durad et al.[26] Expander	Donor GTs: 3/10 1a 1/10 1a/3 2/10 2 4/10 ND	**GT 1,4 or unknown:** Pre-operative: - EBR/GZR x 1 dose Post-transplant: - EBR/GZR x 12 weeks, (16 weeks + RBV if NS5A RAS occurred) **GT 2,3:** Pre-operative: - EBR/GZR x 1 dose Post-transplant -EBR/GZR + SOF x 12 weeks	1 month	10	30%	preemptive	Induction: rATG Maintenance immunosuppression: Tac MMF prednisone	100%	Median eGFR at 6 months 63.5 mL/min/1.73 m2 (50%) anti-HCV seroconversion at 6 months 40% DGF 10% aminotransferase elevatio*n* > 5x ULN median KDPI score 45% Median VL was 62 400 IU/mL
Molnar et al.[47]	KTRs Genotypes: 34/53 1a 1/53 1 b 3/53 2 15/53 3	Introduced postoperatively and continued for 12 (5 required 16) weeks: GLR/PIB 89% SOF/VEL 9% SOF/LED		53	100%	76 POD	Induction: rATG Maintenance immunosuppression: Tac MMF prednisone	100%	Mean eGFR at SVR12: 67 mL/min/1,73m^2^ 100% patient and graft survival Median follow-up 8 months 7,5% AR, −16/53 DSA 30% (13% class I, 23% class II) −18/53BK viremia 34%, −32/53 CMV viremia −1/53 fibrosing cholestatic hepatitis, −19% AST, 15% ALT elevatio*n* > 3 times normal −3/53 DGF Mean KDPI : UNOS allocation 51% ‘optimal’ 24%
Kapila et al.[46]	Recipients’ Genotypes: 1a 38/64, 1 b 1/64, 1 3/64 2 6/64 3 8/64 4 3/64 1a/3 1/64 2/3 1/64	Introduced postoperatively and continued for 12 weeks: GLE/PIB (60%) (1 patient received SOF/LED for 4 weeks and was transitioned to GLE/PIB for 8 additional weeks) SOF/LED For 12 weeks	23,5 d	64 KTRs	95% 58 of 61 started on DAA’s	72 POD	Induction: rATG Maintenance immunosuppression: Tac MMF prednisone	71% 10 undetectable HCV RNA not eligible for SVR12 7 remains on treatment 1 nonresponder	Patient survival 98% (1 patient died 77 days after transplant due to unknown cause, did not received DAAs treatment) Median follow-u*p* ∼10 months 2 FCH Median KDPI: 54%
Sise et al.[37] MYTHIC	Donor GTs: 1a 13/15 2 1/15 4 1/15	Introduced postoperatively and continued for 8 weeks: GLE/PIB	6.3 weeks	30	79%	2-5 POD	Induction rATG 97% (one rATG + basiliximab) no antibody induction therapy Maintenance immunosuppression (90%): Tac MMF prednisone	100%	Median eGFR at 6 months 57 mL/min/1.73 m^2^ at 6 months Median follow-up 9 months 97% patient and graft survival 1 death attributed to sepsis 9 months post-transplant Median KDPI 53% 10% ACR 10% BKV 23% DGF 17% proteinuri*a* > 1 g/d Median VL_peak_ was 2135 IU/ml
Durad et al.[38]	Donor Genotypes: 1 a 6/10 1 b 1/10 2 2/10 indeterminate 1/10	Single dose preoperativel*y* + 4 weeks post-transplant: GLE/PIB	24 D	10	50%	preemptive		100%	Median follow-up 12 months Median eGFR 54.5 mL/min/1.73 m2 at POW12, One graft failure due to venous thrombosis 100% patient survival No serious treatment-related adverse events were reported No AR reported Median KDPI 60%
Frebius-Kardasz et al.[48]	Donor Genotypes: 1a 2/7 1 b 2/7 3a 1/7 Unknown 2/7	Introduced postoperatively and continued for 8/12 weeks: SOF/LED +/- RBV SOF/VEL		7	100%	POD 7	Induction baziliximab (+PEX/IVIG in one patient) Maintenance immunosuppression Tac (85%), MMF prednisone	100%	Mean eGFR 63 mL/min/1.73m^2^ at SVR No AR reported No serious treatment-related adverse events were reported Median VL_peak was_ 291 500 IU/mL
Forbes et al.[40]	Recipients GT: 1a 7/12 1a/1b 2/12 3 3/12	Introduced postoperatively and continued for 12 weeks: GLE/PIB(75%) SOF/LED *Perioperative: 6/12 kidneys pumped with perfusate exchange 6/12 paired kidneys without intervention	58.7 D	12	100%	32 POD pumped cohort 26 POD unpumped cohort	Induction: Alentuzumab 92% Basiliximab Maintenance immunosuppression: TAC MMF Prednisone	50% SVR 12 (6 pending) 100% SVR4	100% patient survival, 92% graft survival Median follow-u*p* ∼6.2 months 1 AMBR resulting in graft loss (received a pumped kidney) average KDPI 47,5% the pumped patient cohort VL was 413 471 IU/ml /unpumped 4 360 359 IU/ml at POW 1

GT = genotype; KTR = kidney transplant recipient; rATG = rabbit antithymocyte globulin; Tac = tacrolimus; GLE/PIB = glecaprevir/pibrentasvir; HCV = hepatitis C virus; NAT = nucleic acid testing; KDPI = kidney donor profile index; MMF = mycophenolate mofetil; eGFR = glomerular filtration rate estimated, Cr = serum creatinine; SOF/LED = sofosbuvir/ledipasvir; SOF/VEL = sofosbuvir/veltapasvir; EBZ/GPR – elbasvir/grazoprevir; SVR 12 = sustained viral response 12 weeks after treatment cessation; BKV = polyoma virus viremia; DGF = delayed graft function; POD – post operating day; AR – acute rejection; ABMR – antibody-mediated rejection; TAC – tacrolimus; MMF – mycophenolate mofetil; D – day; ULN – upper limit of normal; KT = kidney transplantation; ICF = Informed Consent Form; RAV = resistance-associated variant; PEX = plasmapheresis; IVIG = intravenous immunoglobulins; ACR = acute cellular rejection.

The EXPANDER study, in turn, utilized prophylactic DAA therapy. One dose of EBR/GZR was administered before transplantation, and the treatment was continued for 12 weeks [26]. For those with genotypes other than 1, sofosbuvir (SOF) was added to EBR/GZR. SVR was achieved in 100% of cases, and severe complications such as AR, graft loss, or liver damage were not observed (Table 1).

The possibility of shortening the treatment duration to below the standard of 12 weeks has also attracted much interest. Sise et al. conducted a study in which glecaprevir/pibrentasvir (GLE/PIB) was administered for 8 weeks starting from the second to fifth day after transplantation [37,38]. Thirty HCV-naïve patients underwent kidney transplantation from HCV-viremic donors and were followed up for a median duration of 9 months. All patients achieved SVR and acute cellular rejection (ACR) and BK viremia were observed in three patients. Durand et al. enrolled 10 patients who received GLE/PIB for 4 weeks, with the first dose being administered before transplantation. Virus transmission was observed in 50% of KTRs, and 100% SVR was observed after 12 weeks. The authors reported 100% patient survival and one graft loss attributed to vein thrombosis. However, they reported no episodes of AR [38].

Gupta et al. presented an even more aggressive strategy of limiting the time of DAA therapy after HCV NAT D+/R- transplantation [43]. In the DAPPeR study, patients received a single dose of DAAs, followed by one or three doses after transplantation. Administering two doses of DAAs resulted in an infection transmission of approximately 30%, while a 4-day protocol helped limit it to 7.5%. Only six patients required treatment, while the rest had low, self-limiting viremia. Ultimately, 83% of the treated patients achieved SVR. One patient, in whom the infection relapsed, did not achieve SVR with the first or second course of treatment. This patient did not wish to receive further treatment. Incidence of ACR and development of *de novo* donor-specific antibodies (DSA) did not exceed those observed in HCV-aviremic donors and accounted for 4% and 6% respectively (Table 1) [44,45].

The breakthrough in transplantation that occurred after the publication of the THINKER and EXPANDER study results encouraged other researchers to attempt replicating these results in ‘real-life’ studies. Kapila et al. presented the largest such study, which included 64 KTRs. Treatment with GLE/PIB or sofosbuvir/ledipasvir lasted 12 weeks and was initiated 72 days after transplantation. Three recipients did not develop viremia, even though the donors had low, but detectable viremia lower than 142 IU/mL. All but one patient achieved SVR. One patient did not respond to treatment because of resistance to NS5A inhibitors and was retreated with sofosbuvir/velpatasvir/voxilaprevir [46]. Fibrosing cholestatic hepatitis was observed in two patients; eleven and fourteen weeks after transplant. Both were successfully treated with DAAs.

Several other real-life and clinical studies have provided satisfactory results in treating HCV from a donor organ (Table 1) [47,48]. Promising results of HCV NAT D+/R- transplants can also be seen in case of other organs, such as the heart or lungs [49,50].

## Willingness to accept HCV-viremic organs

A study by Potluri et al. showed that over the period from 2015 to 2019, the willingness to accept a seropositive organ increased sixfold [16]. However, other studies point to the fact that recipients infected with HCV are unwilling to accept an infected graft. This results in a great majority of HCV NAT + kidneys being transplanted to HCV-naïve recipients [16]. An analysis by McCauley et al. showed that as many as 80% of patients are willing to accept an organ from an HCV NAT + donor under certain conditions, while 18% would not accept it under any circumstance [51]. This decision is mainly influenced by the expected effectiveness of treatment, quality of the organ, and the duration of being on the organ waiting list. The above study also points to a lack of knowledge among patients about HCV infection. This underscores the need for comprehensive education programs and access to reliable data to help KTRs make an informed decision regarding HCV NAT D+/R- transplants.

## Donor/recipient selection criteria

HCV NAT D+/R- transplants require careful donor and recipient selection. However, standards governing which patients could benefit from receiving an organ from an HCV NAT + donor and which donors should be considered as potential candidates are currently lacking. Similarly, no unified regulations exist regarding the quality of organs obtained from HCV NAT + donors.Unquestionably, patients with long anticipated waiting times should be considered for HCV NAT D+/R- transplants if this may reduce the waiting time. Their condition is likely to deteriorate, or they may die, until an organ becomes available; hence, HCV NAT D+/R- transplantation confers a survival benefit to these patients. Moreover, for these patients, remaining on the waitlist may constitute a greater risk than being infected with HCV that may be successfully treated in great majority of cases.

A survey by Lentine et al. showed that HCV-naïve patients with cirrhosis or a history of liver disease are not offered HCV-viremic organs [18]. However, even individuals without already diagnosed liver diseases are at risk. More than half of the patients awaiting kidney transplantation are diabetic or prediabetic; similarly, the impact of obesity in this population is higher than that in the general population [52]. Both diabetes mellitus and obesity, which are components of metabolic syndrome, may affect liver function. This is reflected by the greater NAFLD prevalence in these populations, which may exceed 50% [53]. Patients with NAFLD may exhibit normal liver enzymes and remain asymptomatic; therefore, they often remain undiagnosed. Offering HCV-viremic organs to patients who are likely to have undiagnosed liver disease poses a threat of progression of liver disease, including hepatocellular carcinoma, which has been observed even in patients with NAFLD without evidence of cirrhosis [54]. Furthermore, KTRs with diabetes and obesity, as well as undiagnosed liver conditions, who receive organs from HCV NAT + donors face a greater risk of metabolic complications, resulting in a higher risk of mortality [55]. Importantly, screening for NAFLD is not recommended in the general population and approaches to screening in high-risk patients vary across guidelines and is not recommended by the American Association for the Study of Liver Disease (AASLD) [56–58]. Therefore, the implementation of FibroScan in standard recipient evaluation prior to HCV NAT D+/R- transplantation could facilitate proper recipient selection.

A strong positive correlation has been reported between RNA levels in organ donors and recipients [41]. Despite this, donor HCV viremia is not routinely reported. Knowledge of viral load could enable identification of individuals at higher risk of HCV-related complications and determination of optimal timing of DAA therapy initiation.

Patients requiring nonstandard immunosuppression or desensitization therapy are frequently excluded from HCV NAT D+/R- protocols. Drug–drug interactions between immunosuppressive agents and DAAs may also translate to underimmunosuppression or decreased efficacy of DAA therapy. Recent experience shows that this may be mitigated by early initiation of pangenotypic treatment. Pangenotypic DAA regimens have limited drug–drug interactions with tacrolimus and may limit the need to adjust the dose of calcineurin inhibitors, even though tacrolimus levels have to be monitored. The lack of reduction in tacrolimus levels mitigates the risk of underimmunosuppression, and hence the risk of *de novo* DSA development and AR. Nevertheless, it is still essential to identify patients at a high risk of AR, such as highly sensitized patients or those requiring retransplantation, and to carefully weigh the risks and benefits of selecting HCV NAT D+/R- organs.

Although the donor genotype and the presence of RAS might be of great importance in patients who are known to be treatment-experienced, they are mainly not known prior to transplantation. Therefore, excluding donors who were previously treated with NS5 inhibitors or those who have relapsed seems a reasonable mean to avoid difficulties in viral clearance after transplantation and a situation when no antiviral treatment will be available for the patient. However, the history of antiviral treatment is often unknown before transplantation.

The donor’s genotype is crucial if there is no access to pangenotypic antiviral agents. However, in the case of pangenotypic drugs, there is still the issue of RAS in relation to NS5A protease inhibitors, especially in genotypes 1a and 3, which can result in prolonged treatment and a worse treatment result [59]. Contrary to the resistance to NS3-4A protease inhibitors, which resolves in a few weeks or months, the resistance to NS5 protease inhibitors can last for a year and can influence the results of recurrent treatment. Importantly, genotypes 1a and 3 are more often seen in PHS criteria increased risk donors, and this fact is often unknown before transplantation [60]. Studies have estimated that up to 4% of patients require changes in DAA therapy [61,62]. Moreover, in some cases, both the first and second lines of treatment did not allow the patient to achieve SVR. However, the American Society of Transplant Surgeons, in its consensus, stated that a lack of knowledge of the genotype or the presence of RAS should not be a contraindication for HCV NAT D+/R- kidney transplantation, provided that treatment can be initiated without delay [12].

Patients who are predicted not to comply with procedures, DAA schedule, and surveillance requirements after transplant should also not be offered HCV-viremic organs. Education of patients potentially willing to accept HCV-viremic organs could promote compliance with posttransplant DAA therapy. A unified policy regarding donor and recipient selection for HCV NAT D+/R- transplantation could encourage broader utilization of HCV NAT + organs.

## DAAs

HCV therapy involves a combination of two or three DAAs that target nonstructural proteins of the HCV and may be delivered with little adverse effect and high efficacy. Pangenotypic DAAs are preferred. The only drawbacks are accessibility to DAAs and the high cost of DAA therapy.

The AASLD and Infectious Diseases Society of America (IDSA) recommend GLE/PIB for 8 weeks or SOF/VEL for 12 weeks for the treatment of HCV-naïve recipients of HCV-viremic organs other than the liver. However, multiple factors should be considered before initiating DAA therapy, especially if pangenotypic agents are unavailable; these factors include any evidence of liver dysfunction, concomitant medications, and to a lesser extent immunosuppression that will be used after transplantation.

Drug-drug interactions must also be accounted for in the DAA therapy selection process to avoid complications, because premature withdrawal of DAA therapy increases the development of drug resistance. Studies have estimated that up to 45% of patients may require an adjustment of the dosage of the calcineurin inhibitor during DAA therapy [63,64]. Another example is patients who require antiepileptic treatment in the period near transplantation, as antiepileptic drugs are not recommended to be taken together with any DAA. A similar situation occurs in the case of high doses of proton pump inhibitors (PPIs; taken twice daily), such as ledipasvir, and this can negatively influence the effectiveness of DAA therapy. However, standard doses of PPIs (corresponding to 20 mg of omeprazole) can be used without adverse events. A great tool facilitating assessment of drug-drug interactions is interaction checker available on https://www.hep-druginteractions.org/checker.

Treatment should also consider the path of drug elimination and kidney function, but this has been subject to change recently. The AASLD/IDSA guidelines have been drafted such that when applying the recommended schemas of treatment for individual patient groups, the dosage need not be adjusted in the case of patients with chronic kidney disease (CKD) or those receiving dialysis^63^. SOF, which is effective in treating genotype 3, is mainly renally excreted. Therefore, it was not recommended for patients with CKD and an estimated glomerular filtration rate (eGFR) < 30 mL/min/1.73 m^2^, but it was approved in November 2019 by the US Food and Drug Administration for use in patients with an eGFR below 30 mL/min/1.73 m^2^ and those who were dialysis-dependent [63].

## Optimal timing of DAA initiation

Currently, there is little doubt that antiviral treatment in HCV NAT + patients can be postponed until after transplantation, in order to allow them to receive a kidney from a donor infected with HCV. However, the optimal time for introducing treatment in such cases of virus transmission from the donor has not yet been established. The new AASLD/IDSA guidelines suggest the initiation of prophylactic treatment or preemptive treatment latest by 7 days after transplantation [63].

Recent studies have adopted various approaches for the introduction of antiviral treatments. Durand et al. in their study began treatment before transplantation, while in the THINKER study, DAAs were introduced after HCV viremia was detected [26,41,42]. Some centers postpone treatment for a couple of weeks until the patient and immunosuppressive treatment are stable enough to avoid the need for early DAA therapy withdrawal. In the study by Molnar et al., DAA therapy was introduced on average 76 days after transplantation [47]. The authors do not claim that this was the intentional time of introducing the treatment, but rather a result of difficulties in obtaining approval for treatment costs from the insurers. This resulted in complications such as fibrosing cholestatic hepatitis or AR. Moreover, higher than expected incidence rate of CMV viremia and BK viremia were observed. However, a national registry-based study by Yazawa et al. did not prove the association between donor-derived HCV viremia and CMV viremia, whereas the more recent study by Molnar et al. found that KTRs undergoing HCV NAT D+/R- transplant may be at increased risk of developing high-level BK viremia [65,66]. In the THINKER and EXPANDER studies, no serious complications were observed in the early posttransplant period [26,41,42]. A recent survey conducted by Lentine et al. showed that the majority of transplant centers initiated DAA therapy after HCV viremia was detected and after hospital discharge [18].

Postponing treatment for a few months may be associated with serious complications. An acute infection with HCV has been associated with the development of fulminant hepatitis or fibrosing cholestatic hepatitis [67]. By intentionally infecting a recipient with HCV, we also risk the development of thrombotic microangiopathy and a series of immunological complications, such as AR or glomerulonephritis (GN) [68]. Studies have reported the development of GN on the 18th day after liver transplantation from a donor with an active HCV infection as well as renal failure requiring dialysis owing to delayed DAA therapy resulting from problems with acquiring approval from the insurance company [69]. The KDIGO recommends, among other things, monitoring proteinuria every 6 months in patients infected with HCV after transplantation, and performing a biopsy if HCV infection occurs. Confirmation of a relapse in the development of *de novo* GN is an indicator of immediate DAA therapy [11]. Despite this, in HCV NAT D+/R- transplantations antiviral treatment initiation in the peri-transplant period deems advisable.

Early introduction of treatment can also limit the exposure to viremia and the development of complications. Patients who start receiving treatment on call to operating room develop viremia less frequently; this decreases the risk for the recipient and, as studies show, allows shortening the duration of DAA therapy and lowering treatment costs [26,41]. In studies that utilized a prophylactic approach rather than initiating treatment after viremia detection, fewer AR episodes were observed. However, the sample size of these studies was small, and further studies are warranted to confirm these findings. On the other hand, prophylactic DAA therapy can expose patients to treatment that they may not require.

Apart from selecting the appropriate time to introduce treatment, the optimum treatment duration after transplantation is also a subject of debate. Many different strategies have been adopted in recent studies. The standard 12- to 16-week treatment duration allowed the achievement of SVR in 100% of patients under both prophylactic and reactive approaches (after HCV viremia is documented) [26,41,42]. Feld et al. enrolled 30 solid organ transplant recipients, including 10 KTRs, and started treatment with GLE/PIB in conjunction with ezetimibe (an HCV entry inhibitor) before transplantation and continued it for 7 days. This allowed for viral transmission of 67%; however, it remained unclear if viral transmission was prevented or cleared rapidly. Nevertheless, SVR was achieved in all patients [36]. Attempts to shorten the treatment period before transplantation and administering only one or two additional doses after transplantation were less than satisfactory [43]. The first-line treatment allowed only 50% of the patients to achieve SVR. Therefore, short-term DAA therapy is currently not recommended outside of clinical trials owing to insufficient data [63].

## Immunosuppression

The optimal immunosuppression scheme for transplants from an HCV NAT + donor to a naïve recipient remains to be determined. An analysis by Bae et al. showed that KTRs infected with HCV have a 20% lower probability of receiving a less effective inductive treatment with interleukin 2 receptor antagonists rather than with anti-thymocyte globulin [70,71], despite HCV infection being a well-recognized factor that increases the risk of AR. This can be partly related to the clinicians’ fear of overimmunosuppression in patients with HCV infection. Immunosuppressive drugs are known to have a permissive effect on HCV replication. Recent studies have shown that patients infected with HCV should receive standard immunosuppression and that depletive induction should not be avoided if there are reasons to introduce it. A retrospective analysis by Luan et al. based on data from the OPTN/Scientific Registry of Transplant Recipients showed that the type of calcineurin inhibitor (cyclosporine vs. tacrolimus) used in maintenance immunosuppression does not influence the mortality rate in patients with HCV Ab+ [72]. These data are from a period when the categorization of HCV Ab+/NAT- and HCV Ab+/NAT + donors was not possible, and the use of mycophenolate mofetil in the same analysis was related to a lower mortality rate (hazard ratio, 0.77; 95% confidence interval, 0.64-0.92; *p* = 0.005) [72].

Non-pangenotypic DAAs impact calcineurin inhibitors trough level; hence pose a risk of underimmunosuppression. Pangonotypic DAAs significantly mitigate this risk. When GLE/PIB is used, for instance, no tacrolimus dose adjustment is required prior to therapy administration; however, the tacrolimus level needs to be monitored.

## KDPI calculation

In the US, the KDPI is used to estimate the expected organ survival rate after transplantation. Many factors are taken into account, including the presence of anti-HCV antibodies, which in turn often leads to an artificial increase in the KDPI by 0.25 on average in comparison to the actual quality of the organ [16,73,74]. This can lead to improper allocation of organs, which is why the issue of removing anti-HCV antibodies from the KDPI assessment has been advocated in some studies. The Kidney Allocation System (KAS) introduced in 2014, which is based on the KDPI, is supposed to better match the quality of the organ to the predicted survival of the recipient. It assumes that an organ with a KDPI of 20% should be transplanted to a recipient with an estimated posttransplant survival score of 20%. However, as a result of the overestimation of the KDPI, the KAS cannot function in accordance with its original goal. Moreover, some donors are classified as having marginal organ quality (KDPI > 85%), while in reality, the quality of their organ is much better [74].

The excellent quality of organs from donors infected with HCV has been proven by Goldberg et al. and Durand et al., who reported KDPI values of 42–45% [26,41]. The analysis by Graham et al. (DAPPeR study) showed a noteworthy difference between the KDPI calculated according to the standard formula and the ‘optimal’ one obtained after removing the anti-HCV antibodies from the calculation (Table 1) [43,75]. With higher index values, the kidneys will be transplanted into recipients with a shorter expected survival. As HCV Ab+/NAT- donors do not constitute a risk of HCV transmission, their KDPI should not be calculated on the basis of the serological status of HCV, and this has been proven by studies that showed a comparable eGFR 6 months after transplantation, which is considered an indicator of the long-term survival of a transplant [76,77].

## Informed consent

The regulations introduced by the OPTN/UNOS require informed consent from the recipient deciding to accept a kidney from a PHS criteria donor. The acceptance of an HCV NAT + organ by an HCV-negative recipient should be preceded by a comprehensive education process, including information on the benefits and risks of such a solution.

The patient needs to be informed that the data regarding the utilization of HCV NAT + organs in HCV-negative recipients is still limited, and that current data are based on studies conducted on small populations. The long-term effects of these transplants are also unknown. The potential recipient should also be provided information on the eventualities of such a transplantation, such as the shorter time of waiting for an organ, shorter time of dialysis, and the resultant lower mortality rate, as well as potential difficulties, virus transmission paths, risk of transmission to family members, possible complications related to virus replication, and the necessity of undergoing antiviral treatment after transplantation. Owing to the relatively high cost of DAA therapy, the patient’s insurance also needs to be verified to ensure it covers the treatment cost. The patient needs to be given ample amount of time to consult with other specialists or family and to formulate questions and obtain answers.

The newest PHS guidelines suggest that the consent to an organ from an HCV NAT + donor should not be on a separate form, as it currently is, but it should rather be included in the standard form of consent for an organ transplantation [34].

## HCV-viremic organ refusal

Given the ever-growing demand for organs that is hard to satisfy, HCV NAT D+/R- transplants may be lifesaving in certain circumstances, despite the inherent risk associated with this practice. This is because despite the imbalance between the demand and supply of kidneys available for transplantation, hundreds of HCV-viremic kidneys are discarded annually [10].

Denying to accept an HCV NAT + organ or not being offered one entails certain consequences, particularly in transplant centers with long waiting times. Compared to accepting an HCV-viremic graft, awaiting an HCV-negative graft may imply a longer waiting time of 12 months [16]. Receiving a graft earlier usually results in a survival benefit and improved quality of life when compared to receiving an HCV-negative graft [78–80]. Most patients awaiting kidney transplantation are dialysis dependent. The time on dialysis increases the risk of death and complications leading to health deterioration [81]. As a consequence of long waiting times, more than a quarter of patients on the waiting list may die while awaiting kidney transplantation, and a proportion of patients will become too sick to qualify for transplantation [82]. Hence, it is important to state it clearly to the patients that they may not survive until they are offered an HCV-negative graft.

Furthermore, the risk of complications may be partly compensated for by a better quality of organ from a younger PHS criteria donor. Potluri et al. in their analysis showed that the kidney function of recipients from HCV NAT + and HCV Ab+/NAT- donors a year after kidney transplantation was comparable to that of recipients of HCV-negative kidneys, despite the worse KDPI in the former group [16].

Utilization of HCV-viremic kidneys increases the total number of transplantations and results in a shorter waiting time for individuals remaining on the waiting list.

## Insurance

Coverage of cost of DAA therapy remains a significant concern for both patients and health care providers and prevents HCV-viremic organs from being fully utilized. As a part of clinical studies, antiviral treatment is provided by pharmaceutical companies, while in real-life scenarios, the costs have to be covered by the patient or their insurance. Data show that the percentage of refusals of requests to cover medical treatment costs, depending on the study, ranges from 20% to 35% and is more common with a public insurer than with a private one [47]. In all cases, a delayed start of treatment should be considered. Insurance companies explain their reason for rejection as the intentional transmission of the virus or the off-label usage of DAAs, as DAAs are registered only for treating chronic hepatitis C; hence, in this case, the patients have to deal with an acute infection for the first 6 months. In addition, some insurance companies require the documentation of viremia, which is not detectable at the time treatment should be initiated before transplantation, and this can result in a refusal to cover the treatment cost. In the case of a nonimmunocompetent patient, the prolonged time before starting treatment can have catastrophic results. Therefore, some authors think that in the absence of an assurance that medical treatment costs will be covered and given the possibility that patients may not be able to personally cover the treatment costs, such solutions should not be considered.

## Cost-effectiveness

The utilization of organs with active HCV viremia is associated with high costs. However, keeping patients on the waiting list is also costly in terms of the need for dialysis or the use of devices aiding cardiac function. The latest analyses show that transplanting HCV NAT + kidneys to HCV-negative recipients can be cost-effective because it could shorten the waiting time by 2 years; however, other analyses show that this period is 11 months [3,83]. It seems that the cost of DAAs will decrease and shortening the duration of DAA therapy will allow an additional reduction in the cost and availability of such treatments.

## Conclusions

It is rational to prioritize the utilization of HCV NAT + organs in recipients already infected with HCV. This entails lower costs, limits the risk of possible complications, and seems more reasonable from an ethical standpoint. Such organs should be offered to HCV-negative recipients only as a secondary choice. However, this is not always possible in everyday practice.

In light of the current knowledge, the transplantation of HCV NAT + kidneys to naïve recipients may constitute a solution to organ shortage. However, such practice entails a risk of complications, especially when combined with the difficulty in providing DAA therapy in the direct posttransplant period and the need for careful donor and recipient selection. Every effort should be made to ensure DAA therapy and reduce the mortality of patients awaiting kidney transplantation.

Extended education programs should also be implemented to increase awareness of HCV infection and HCV NAT D+/R- kidney transplantation among candidates. This may encourage the acceptance of HCV-viremic organs and simultaneously maximize compliance and mitigate the risks associated with this procedure. Currently, it seems premature to utilize HCV NAT D+/R- kidney transplantation as a standard of care. Further studies are required to draw solid conclusions regarding the long-term consequences of adopting such a treatment approach.
